# Insights into embryonic chromosomal instability: mechanisms of DNA elimination during mammalian preimplantation development

**DOI:** 10.3389/fcell.2024.1344092

**Published:** 2024-02-05

**Authors:** Jacqueline Budrewicz, Shawn L. Chavez

**Affiliations:** ^1^ Department of Molecular and Medical Genetics, Oregon Health and Science University, Portland, OR, United States; ^2^ Division of Reproductive and Developmental Sciences, Oregon National Primate Research Center, Beaverton, OR, United States; ^3^ Department of Obstetrics and Gynecology, Oregon Health and Science University, Portland, OR, United States; ^4^ Department of Biomedical Engineering, Oregon Health and Science University, Portland, OR, United States

**Keywords:** cellular fragmentation, DNA shedding, excluded blastomere, micronuclei, mitosis, nuclear budding, preimplantation embryo, aneuploidy

## Abstract

Mammalian preimplantation embryos often contend with aneuploidy that arose either by the inheritance of meiotic errors from the gametes, or from mitotic mis-segregation events that occurred following fertilization. Regardless of the origin, mis-segregated chromosomes become encapsulated in micronuclei (MN) that are spatially isolated from the main nucleus. Much of our knowledge of MN formation comes from dividing somatic cells during tumorigenesis, but the error-prone cleavage-stage of early embryogenesis is fundamentally different. One unique aspect is that cellular fragmentation (CF), whereby small subcellular bodies pinch off embryonic blastomeres, is frequently observed. CF has been detected in both *in vitro* and *in vivo*-derived embryos and likely represents a response to chromosome mis-segregation since it only appears after MN formation. There are multiple fates for MN, including sequestration into CFs, but the molecular mechanism(s) by which this occurs remains unclear. Due to nuclear envelope rupture, the chromosomal material contained within MN and CFs becomes susceptible to double stranded-DNA breaks. Despite this damage, embryos may still progress to the blastocyst stage and exclude chromosome-containing CFs, as well as non-dividing aneuploid blastomeres, from participating in further development. Whether these are attempts to rectify MN formation or eliminate embryos with poor implantation potential is unknown and this review will discuss the potential implications of DNA removal by CF/blastomere exclusion. We will also extrapolate what is known about the intracellular pathways mediating MN formation and rupture in somatic cells to preimplantation embryogenesis and how nuclear budding and DNA release into the cytoplasm may impact overall development.

## Introduction

Chromosomal instability (CIN) is a common form of genomic variability that causes changes in chromosome number and/or structure (Bielski and Taylor, 2021). It is characterized by either whole or segmental duplications/deletions of individual chromosomes or produces complex abnormalities that impact multiple chromosomes simultaneously. Losses and/or gains of entire chromosomes is termed aneuploidy and results in copy number variation (CNV) between cells. Besides containing a different number of chromosomes than their chromosomally normal (euploid) counterparts, aneuploid cells will also likely experience gene dosage differences that manifests as genetic variation (Kojima and Cimini, 2019; Stamoulis et al., 2019). While thought to be hallmarks of tumor transformation and cancer, CIN and aneuploidy are also frequently observed in mammalian preimplantation embryos (Vanneste et al., 2009). Aneuploidy can arise in the gametes during meiosis or after fertilization from the mitotic divisions of early embryogenesis. Unlike mitotic divisions in somatic cells, however, cleavage-stage embryos increase in cell number without a change in overall size. This largely occurs in the absence of new transcription until the major wave of embryonic genome activation (EGA) around the ∼4–8 cell stage in several mammals (Braude et al., 1988; Dobson et al., 2004; Vassena et al., 2011; Asami et al., 2022). Thus, the first three mitotic divisions are the most susceptible to chromosome mis-segregation events. Depending on the stage at which it arises, a mitotic error can be just as detrimental as a meiotic error and induce embryo arrest or be surmounted in subsequent development. Even if an aneuploid embryo reaches the blastocyst stage, however, it can still fail to implant or result in spontaneous miscarriage following implantation (Hassold et al., 1980; Schaeffer et al., 2004).

**FIGURE 1:**
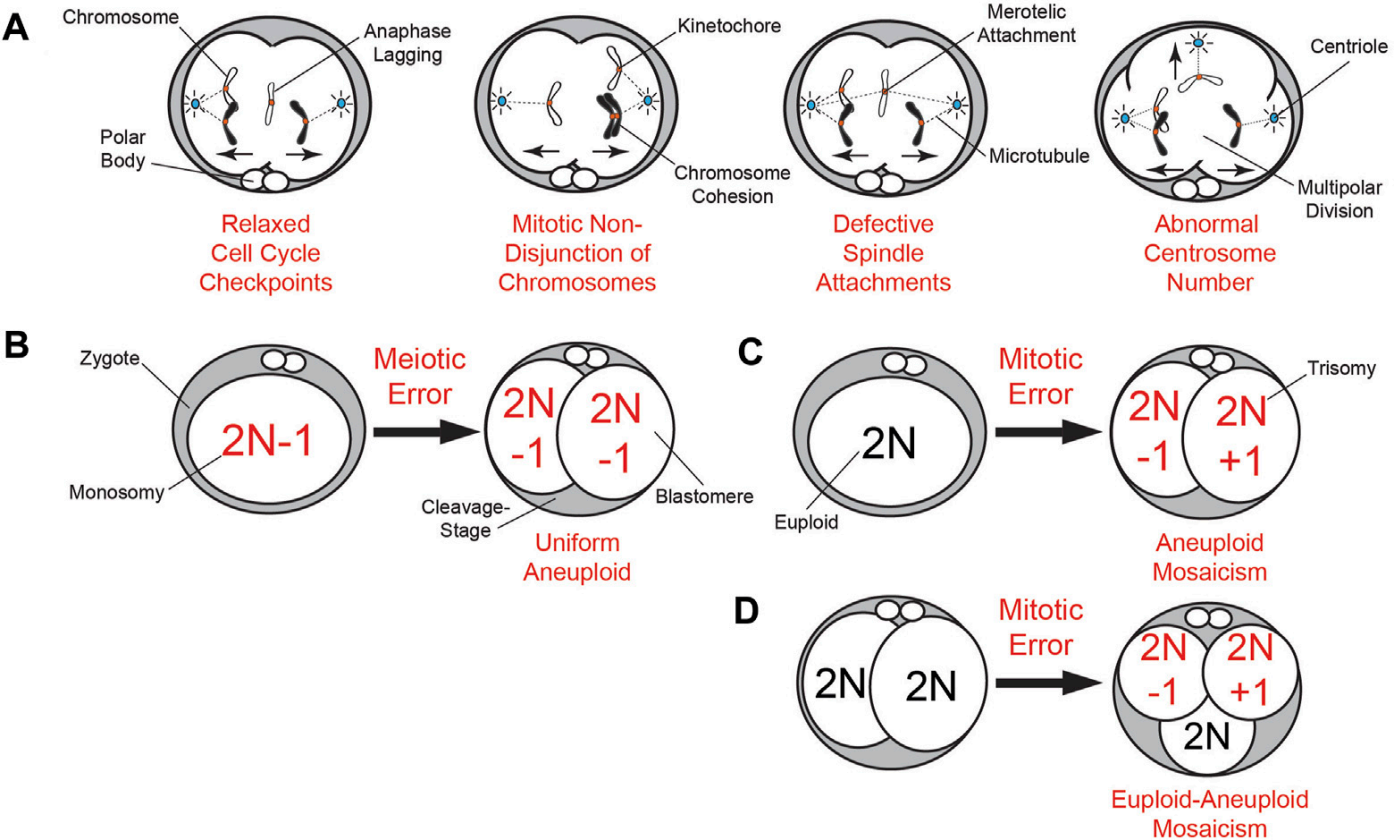
Summary of meiotic and mitotic chromosome mis-segregation mechanisms and embryonic aneuploidy outcomes. **(A)** Known mechanisms of mitotic mis-segregation from dividing somatic cells that have also been detected to some extent in cleavage-stage embryos from humans (Coonen et al., 2004), NHPs (Daughtry et al., 2019), and livestock (Yao et al., 2018; Brooks et al., 2019; Brooks et al., 2022). This includes relaxed cell cycle checkpoints that permit aberrations such as anaphase lagging of chromosomes, non-disjunction events due to premature loss or prolonged chromosome cohesion, defective spindle attachments of chromosomes to microtubules, and abnormal centrosome number leading to multipolar divisions. **(B)** The inheritance of a meiotic segregation error (a monosomy in this case) from the oocyte results in a 2-cell embryo with uniform aneuploidy, whereby each daughter blastomere has the same karyotype (2N−1). **(C)** Mitotic chromosome mis-segregation in a zygote during the first cleavage division can be just as detrimental as the inheritance of a meiotic error, producing a 2-cell embryo with blastomeres exhibiting different chromosomal losses (2N−1; monosomy) or gains (2N + 1; trisomy) known as aneuploid mosaicism. **(D)** Mitotic errors occurring later in development beyond the 2-cell stage produce embryos with euploid-aneuploid mosaicism, or those containing a mixture of both euploid and aneuploid blastomeres.

Our knowledge of mitotic chromosome mis-segregation largely derives from somatic cells and includes relaxed cell cycle checkpoints, premature loss or prolonged chromosome cohesion, defective spindle attachments, and abnormal centrosome number (Ganem et al., 2009; Soto et al., 2019) (Figure 1A). However, only the mitotic checkpoint complex (MCC) has been investigated in greater detail within the context of embryogenesis (Wei et al., 2011; Bolton et al., 2016; Vazquez-Diez et al., 2019a; Brooks et al., 2022). At the zygote stage, replication stress due to incomplete DNA replication and early entry into mitosis also contributes to chromosome mis-segregation (Palmerola et al., 2022). Following a mis-segregation event, the chromosome(s) will become encapsulated in nuclear envelope (NE) and form abnormal intracellular structures known as micronuclei (MN) that are spatially distinct from the main nucleus (Chavez et al., 2012; Daughtry et al., 2019) . In response to micronucleation, a dynamic process called cellular fragmentation (CF), whereby small extracellular units pinch off from blastomeres, has been shown to occur and is unique to preimplantation development (Antczak and Van Blerkom, 1999). We have also demonstrated that whole chromosomes or chromosomal segments may be sequestered into CFs (Chavez et al., 2012; Daughtry et al., 2019), but whether this originated from MN or was produced from a different process remains unknown. Because they lack or have defective NE, the chromosomal material contained within MN and CFs becomes highly susceptible to DNA breakage. Despite this damage, embryos may continue to divide and exclude any CFs, as well as non-dividing blastomeres, from participating in blastocyst development (Daughtry et al., 2019). Upon blastocyst expansion, the placental-derived trophectoderm (TE) layer becomes mechanically constrained, causing the release of DNA into the cytoplasm (Domingo-Muelas et al., 2023). In this review, we will discuss how DNA elimination via CF and blastomere exclusion could impact the overall chromosome composition of embryos and what is known about MN rupture and DNA loss in somatic cells and early embryogenesis.

## Meiotic *versus* mitotic origin(s) of aneuploidy in mammalian preimplantation embryos

Using whole-genome approaches, it was shown that 50%–80% of even high-quality cleavage-stage human embryos are aneuploid to some extent (Vanneste et al., 2009; Johnson et al., 2010b; Chavez et al., 2012; Chow et al., 2014; McCoy et al., 2015), which is major contributor to *in vitro* fertilization (IVF) failure. Estimates of aneuploidy in human blastocysts based on TE biopsy are less than the cleavage-stage, but still quite high at 20%–40% (Johnson et al., 2010b; Fiorentino et al., 2014; Popovic et al., 2018). By inferring chromosome dosage from single-cell RNA-seq data, an analysis of the morula-to-blastocyst transition suggested that the aneuploidy frequency at this stage of development is probably closer to 80% (Starostik et al., 2020). Indeed, a recent study used single-cell DNA-seq (scDNA-seq) of human blastocysts to demonstrate numerical and structural chromosome abnormalities in 82% of the embryos (Chavli et al., 2024). A similarly high percentage of aneuploidy is thought to occur in cleavage-stage embryos from rhesus monkeys (Daughtry et al., 2019), but to a lesser extent in bovine (Destouni et al., 2016; Hornak et al., 2016; Tsuiko et al., 2017) and porcine embryos (Hornak et al., 2015), and 1%–4% of murine embryos (Macaulay et al., 2015; Treff et al., 2016). To date, only DNA-Fluorescence *In Situ* Hybridization of a subset of chromosomes has been used to assess the frequency of aneuploidy in cleavage-stage equine embryos (Rambags et al., 2005). However, like bovine oocytes, equine oocytes are typically matured *in vitro*, which is known to increase aneuploidy by itself (Treff et al., 2016). Chromosomal mis-segregation in oocytes that arises during meiosis is one of the primary sources of aneuploidy and estimated to be around ∼20% in young women, but this percentage increases with maternal age (Hou et al., 2013; Gruhn et al., 2019; Charalambous et al., 2022) (Figure 1B). However, there is evidence that these meiotic errors can be corrected during oocyte maturation by the extrusion of reciprocal aneuploid polar bodies (PBs) (Hassold and Chiu, 1985; Forman et al., 2013; Treff et al., 2016). In contrast, sperm typically exhibit an aneuploidy frequency of ∼1–2% that is not influenced by paternal age (Lu et al., 2012; Bell et al., 2020). Although specific factors to meiotic mis-segregation have been identified, much of our knowledge of mitotic mis-segregation comes from somatic cells during tumorigenesis and cancer progression. This is in spite of findings that mitotic errors in cleavage-stage embryos are just as, or more, frequent as meiotic errors and appear independent of both maternal age or fertility status (Vanneste et al., 2009; Chavez et al., 2012; McCoy et al., 2015) (Figures 1C, D).

## Mechanisms of chromosome mis-segregation during gametogenesis and embryogenesis

Non-disjunction, or the failure of homologous chromosomes (meiosis I) or sister chromatids (meiosis II) to properly separate, is the most common cause of chromosome mis-segregation in oocytes (Wartosch et al., 2021) (Figure 1B). While non-disjunction can occur during both meiotic stages, non-disjunction events leading to major aneuploidy issues such as uniform monosomy or trisomy originate primarily from meiosis I (Ghosh et al., 2009; Herbert et al., 2015). Mitotic non-disjunction is also observed in embryos following fertilization, but the failure of parental chromosomes to properly migrate to the spindle poles during anaphase is thought to be the most frequent mechanism for aneuploidy to arise during cleavage divisions (Coonen et al., 2004; Daughtry et al., 2019; Brooks et al., 2022) (Figure 1A). However, anaphase lagging of chromosomes in somatic cells is often indicative of defects in kinetochore-microtubule attachments and therefore, these are not necessarily mutually exclusive events (Thompson and Compton, 2011). There is also evidence that incomplete DNA replication during the S phase of the cell cycle and premature entry into mitosis results in subsequent whole and segmental chromosome errors at the zygote stage (Palmerola et al., 2022).

By monitoring the bipolar attachment of spindle microtubules to kinetochores, the MCC prevents activation of the anaphase promoting complex/cyclosome and delays mitotic progression until stable attachments are established. The main components of the MCC are evolutionarily conserved and include CDC20, as well as the serine/threonine kinases, BUB1B/BUBR1, BUB3, and MAD2. Unlike somatic cells, which require all three kinases to prevent aneuploidy, gametes and embryos primarily rely on BUBR1/BUB1B to ensure chromosome fidelity (Jeganathan and van Deursen, 2006; Touati et al., 2015; Vazquez-Diez et al., 2019b; Brooks et al., 2022). Moreover, because Bub1B/BubR1 has been shown to be maternally inherited in *Drosophila* (Perez-Mongiovi et al., 2005) and early cleavage divisions are largely under the control of maternal protein and RNA until EGA (Braude et al., 1988; Dobson et al., 2004; Vassena et al., 2011; Asami et al., 2022), BUBR1/BUB1B and other cell cycle associated factors derived from the oocyte likely contribute to maternal age related fertility decline (Qiu et al., 2018). Sperm also provide a small number of transcripts at fertilization, but whether these RNAs participate in EGA or other processes during the oocyte-to-embryo transition is still under investigation (Sendler et al., 2013; Corral-Vazquez et al., 2021). However, it has been shown that certain sperm borne microRNAs regulate early mitotic timing in human embryos and are required for the first cleavage division at least in mice (Liu et al., 2012; Shi et al., 2020). The centrosome for the first mitotic division(s) is also paternally inherited from the sperm in most mammalian species except rodents and can still cause aneuploidy via abnormal divisions (Sathananthan et al., 1991; Schatten et al., 1991) (Figure 1A).

### CF is likely a response to chromosome mis-segregation and micronuclei formation

**FIGURE 2:**
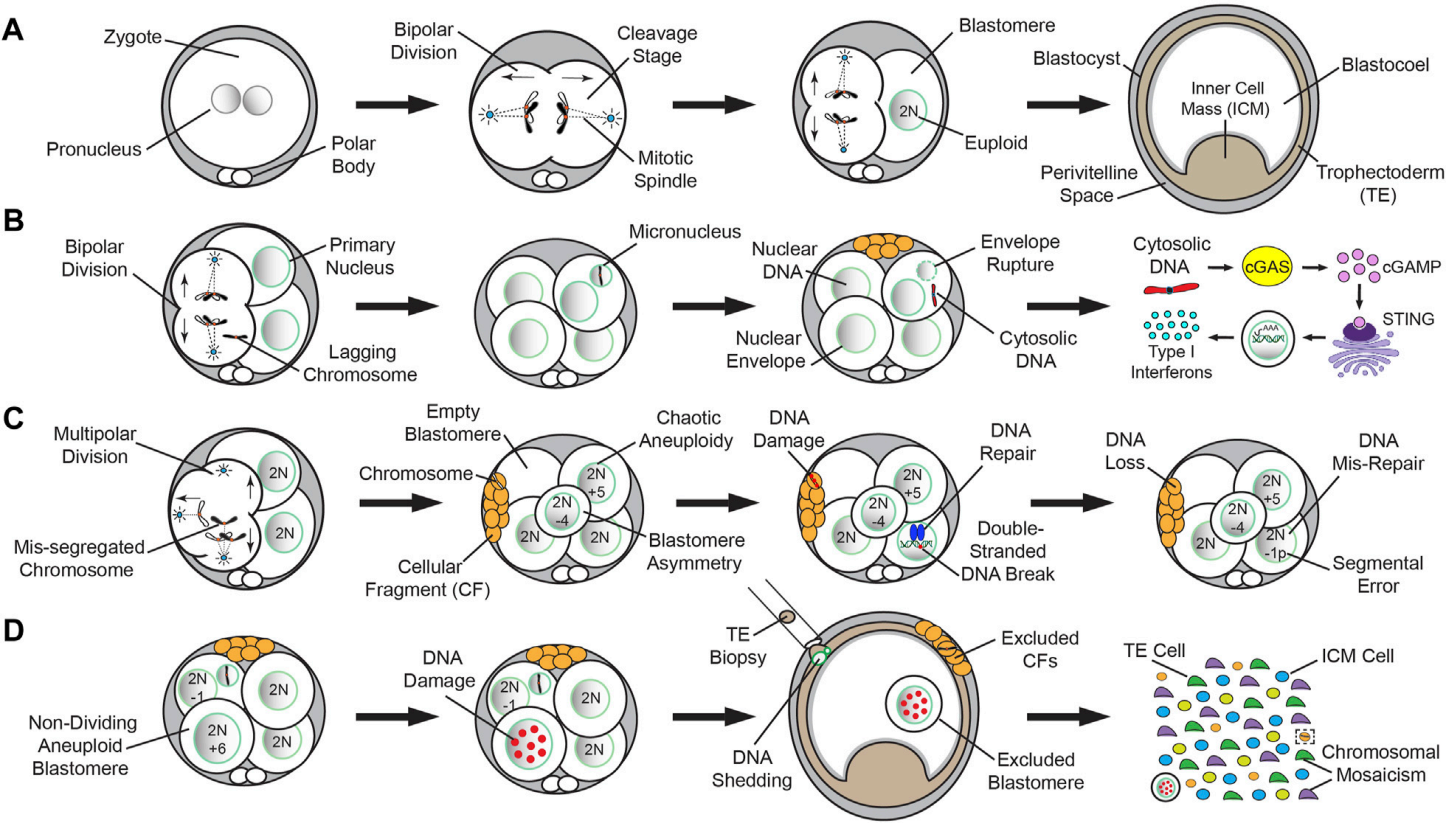
Normal versus abnormal mitotic chromosome segregation and different mechanisms of DNA loss from embryos. A simplified model of normal mitotic chromosome segregation in mammalian cleavage-stage embryos and mis-segregation induced DNA elimination up to the blastocyst stage in humans (Chavez et al., 2012; Domingo-Muelas et al., 2023), NHPs (Daughtry et al., 2019), horses (Brooks et al., 2019), and cattle (Yao et al., 2018; Brooks et al., 2022; Yao et al., 2022). **(A)** Normal mitotic chromosome segregation events from bipolar cleavage divisions are more likely to produce euploid embryos that reach the blastocyst stage. **(B)** A segregation error from anaphase lagging or another mechanism during mitosis can still result in the formation of MN. The appearance of MN in embryos is likely a precursor to CF since MN may be detected in the absence of CF, but not *vice versa*. MN are fragile and their nuclear envelope often ruptures, leading to the release of DNA into the cytoplasm. In other biological contexts, cytosolic DNA activates the cGAS-STING DNA-sensing pathway, which in turn, induces an immune response through transcription of Type I Interferons (IFNs) and other cytokines. **(C)** Multipolar divisions at the cleavage-stage often result in blastomere asymmetry, chaotic aneuploidy, and/or one or more cells lacking a nucleus (empty blastomere). Although there are likely other mechanisms, CF during or following the abnormal division may capture whole chromosomes or chromosomal segments lost from blastomeres. The chromosome(s) contained within CFs lack a nuclear envelope and undergo DNA damage, resulting in eventual DNA loss. In contrast to whole chromosomal mis-segregation, segmental errors most often emerge from improperly repaired double-stranded DNA breaks. **(D)** Despite the presence of MN, CF, and/or non-dividing aneuploid blastomeres, certain cleavage-stage embryos will still successfully reach the blastocyst stage. However, these entities are often subjected to extensive DNA damage. Upon blastocyst formation, chromosome-containing CFs may be expelled to the perivitelline space, while the arrested blastomeres are excluded into the blastocoel cavity. Blastocyst expansion mechanically constrains the TE layer, causing nuclear budding and the shedding of DNA into the cytoplasm, which is exacerbated by TE biopsy. Single-cell analyses of disassembled blastocysts and co-isolation of DNA and RNA from the same individual cell will assist in determining the impact of chromosome loss on the embryo and the extent of chromosomal mosaicism between the TE and ICM when cell identity is confirmed by gene expression.

Even though bipolar divisions are more likely to produce euploid embryos that reach the blastocyst stage (Figure 2A), a segregation error from anaphase lagging or another mechanism during mitosis can still result in the formation of MN (Figure 2B). MN are frequently detected in early cleavage-stage embryos from humans (Chavez et al., 2012), nonhuman primates (NHPs) (Daughtry et al., 2019), horses (Brooks et al., 2019), and cattle (Yao et al., 2018; Brooks et al., 2022; Yao et al., 2022), but not in murine embryos at comparable levels until the morula stage (Vazquez-Diez et al., 2016; Vazquez-Diez et al., 2019b). Once formed, MN can sustain one of multiple fates, including persistence in subsequent divisions, fusion with the primary nucleus from which it arose, or relocation to an adjacent cell via a chromatin bridge during anaphase (Vazquez-Diez et al., 2016; Brooks et al., 2022). The appearance of MN in embryos is likely a precursor to CF since MN may be detected in the absence of CF, but not *vice versa* (Daughtry et al., 2019). Analogous to aneuploidy, the incidence of CF varies across mammalian species, with ∼50% of human, NHP, equine, and porcine embryos exhibiting CF (Alikani et al., 1999; Antczak and Van Blerkom, 1999; Tremoleda et al., 2003; Mateusen et al., 2005; Daughtry et al., 2019), ∼15% of bovine embryos undergoing CF (Somfai et al., 2010; Sugimura et al., 2010), and murine embryos rarely displaying CF unless experimentally induced (Dozortsev et al., 1998; Chavez et al., 2014). CF has been shown to occur *in vivo* in several mammals, including humans (Pereda and Croxatto, 1978; Buster et al., 1985; Gjorret et al., 2003; Mateusen et al., 2005), indicating it is not an artifact of *in vitro* culture, and is distinct from the cell death-induced DNA fragmentation that can arise late in preimplantation development (Hardy et al., 2001; Xu et al., 2001). Despite indications that CF is evolutionary shared among some mammals, comparative studies focused on the mechanistic details of CF and its relationship to aneuploidy are still lacking.

### Whole chromosomes and/or segmental DNA may be contained within CFs

While CF was first described in the 1980s as a morphological indicator of an embryo with low implantation potential (Trounson and Sathananthan, 1984; Alikani et al., 1999), the significance of CF and how it impacted embryogenesis was only recently determined. In 1999, the timing and pattern of CF in human embryos was examined and the authors concluded that the earlier in preimplantation development CF occurred, the worse the outcome (Antczak and Van Blerkom, 1999). Four basic patterns of CF were also proposed: (1) a single monolayer of small CFs on the surface of one blastomere with no apparent reduction in cell size, (2) the presence of multiple layers of CFs accompanied by a significant reduction in blastomere size, (3) complete disintegration of one or more blastomeres into CFs, and (4) a limited number of small CFs scattered over several blastomeres with no indication of which cell(s) they came from. The findings also suggested that the release of large fragments at the early cleavage-stage may deprive blastomeres of vital proteins and organelles, hindering further development. Initial attempts to selectively remove CFs by microsurgery suggested that this procedure improved blastocyst formation and IVF outcomes (Eftekhari-Yazdi et al., 2006; Keltz et al., 2006), but other studies reported no benefit from fragment removal (Halvaei et al., 2016). Ultrastructure analyses of both *in vitro* and *in vivo* derived embryos revealed that mitochondria were the most abundant organelle in CFs (Pereda and Croxatto, 1978; Halvaei et al., 2016), and immunostaining suggested that there was DNA in some fragments (Chavez et al., 2012). It was not until the contents of CFs were examined by next-generation sequencing and the entire embryo reconstructed at a single-cell level, however, that this was confirmed. Using rhesus macaque embryos as a model for human preimplantation development, our group demonstrated that fragments may encapsulate whole chromosomes or chromosomal segments lost from blastomeres (Daughtry et al., 2019) (Figure 2C). There was no preferential sequestering of certain chromosomes as both small and large chromosomes were affected, and high variability in the size of DNA segments, with a slightly greater propensity for maternal chromosomes to be encapsulated. However, while ∼18% of the embryos had chromosome-containing fragments, only ∼6% of all CFs contained DNA detectable by sequencing. Given that most embryos with CF exhibited non-reciprocal chromosome losses not found in other cells or fragments (Daughtry et al., 2019), this suggested that CF is not efficient at removing mis-segregated chromosomes or there is an alternative fate for MN.

### Time-lapse imaging uncovers the mechanism(s) of chromosome encapsulation by CFs

Although many aneuploid embryos will arrest at the cleavage-stage, some will still form blastocysts and may even appear morphologically indistinguishable from euploid embryos, especially when assessed at static time points (Magli et al., 2000; Dupont et al., 2010). Thus, the use of time-lapse imaging to monitor preimplantation embryogenesis in real-time has enabled the tracking of morphological events indicative of developmental potential such as the timing, polarity, and symmetry of mitotic divisions, as well as the fate of CFs (Daughtry and Chavez, 2018). Time-lapse imaging has also shown that a fragment can be reabsorbed by the original blastomere from which it arose, providing an opportunity to restore euploidy (Chavez et al., 2012). If a chromosome-containing fragment fuses with a neighboring blastomere that is euploid, however, this could have detrimental effects on the embryo (Hardarson et al., 2002). When we evaluated whether there were imaging parameters indicative of chromosome sequestration by CFs in our study, there was a clear correlation between this event and multipolar divisions at the zygote or 2-cell stage (Daughtry et al., 2019). Consequently, the multipolar division often resulted in blastomere asymmetry, chaotic aneuploidy, and/or one or more cells lacking a nucleus (Figure 2C). In extreme cases of chaotic aneuploidy, the multipolar division produced embryos where every cell had multiple chromosomes affected in what appeared to be a random pattern. Not all embryos with chromosome-containing CFs exhibited multipolar divisions, however, suggesting that there are other mechanisms leading to chromosome encapsulation and potential loss.

## Mitotic mis-segregation events often lead to chromosomal mosaicism

Unlike mitotic errors at the zygote stage (Figure 1C), a mitotic mis-segregation event that arises in 2-cell embryos or later in development can produce cells with diverse karyotypes known as chromosomal mosaicism (Figure 1D). Euploid-aneuploid mosaic embryos containing a mixture of both chromosomally normal and abnormal cells are the most common (Chuang et al., 2020), but there are also reports of embryos with mixoploidy, whereby cells differ according to whether they are haploid, diploid, or polyploid (Destouni et al., 2016; Carson et al., 2018; Daughtry et al., 2019; De Coster et al., 2022). However, these types of errors typically go undetected and/or are classified as euploid unless parental DNA is inputted to assign chromosomes as either maternal or paternal. While it has been known for quite some time that the smaller chromosomes are more susceptible to mis-segregation events (Smith et al., 1998), recent studies suggest that mosaicism disproportionately impacts large chromosomes (Chuang et al., 2020). Chromosomal rearrangements also often emerge that were produced from improper repair of double-stranded DNA breaks (Burssed et al., 2022) (Figure 2C). This mis-repair leads to DNA damage and segmental errors that may still impact implantation potential, resulting in a higher likelihood of miscarriage and reduced live birth rate if transferred (Zore et al., 2019). Several studies have now shown that segmental aneuploidy is more often mitotic in origin, mostly paternally-derived, and tends to occur within distinct chromosomal regions (Chavez et al., 2012; Babariya et al., 2017; McCarty et al., 2022; Palmerola et al., 2022). Regardless of whether whole and/or partial chromosomes are affected, the DNA will become subjected to further damage due to the fragility of the NE that encapsulates the mis-segregated material, which can have significant consequences for embryo development.

### MN rupture and the release of cytosolic DNA is irreversible and potentially catastrophic

Studies in cancer cells have shown that the NE of MN is quite fragile for a variety of reasons, including premature chromatin condensation in MN, asynchrony in the timing and rate of DNA replication between MN and the primary nucleus, and the failure of MN to import key proteins such as nuclear pore complexes necessary to maintain NE integrity (Xu et al., 2011; Crasta et al., 2012; Liu et al., 2018). This eventually results in MN rupture (Figure 2B), leading to the irreversible loss of nuclear-cytoplasmic compartmentalization and a greater propensity for chromothripsis to occur (Hatch et al., 2013). Chromothripsis is a catastrophic mutagenesis process, whereby chromosomes are shattered and randomly reassembled, promoting somatic cell tumorigenesis and cancer genome evolution (Cortes-Ciriano et al., 2020; Shoshani et al., 2021). Similar events are also known to arise in the germline of patients with certain congenital disorders and there are strong indications of a paternal bias in its origin (Kloosterman and Cuppen, 2013). While hallmarks of this process have been observed during early embryogenesis (Pellestor, 2014; Pellestor et al., 2014), whether preimplantation embryos are equally afflicted by chromothripsis is difficult to determine given the depth of genome coverage and large amplicon size needed to accurately call such small structural variants.

Upon nuclear rupture in other biological contexts, the genetic material contained within MN is released into the cytoplasm, resulting in the activation of the cyclic GMP-AMP (cGAS)-cyclic GMP-AMP receptor stimulator of interferon genes (STING) DNA-sensing pathway. Although cGAS typically resides in the cytoplasm, it can also be observed in the nucleus and plasma membrane (Barnett et al., 2019; Volkman et al., 2019), suggesting that its subcellular location may confer specificity. STING is normally present in the endoplasmic reticulum, but transferred to the Golgi after activation, where it activates the TANK-binding kinase 1 (TBK1) (Mukai et al., 2016; Ogawa et al., 2018). In turn, TBK1 phosphorylates interferon regulatory factor 3 and nuclear factor kappa-light-chain-enhancer of activated B cells (NFκB) (Yum et al., 2021), which translocate from the cytoplasm to the nucleus and induce transcription of type I Interferons and other cytokines (Sun et al., 2013; Wu et al., 2013). As part of the innate immune response, cGAS-STING typically provide protection from the invasion of pathogenic DNA during viral or microbial infections (Mackenzie et al., 2017; Yu and Liu, 2021). However, the cGAS-STING pathway can also be activated by the presence of endogenous DNA produced from chromosomal instability in tumorigenesis and cancer progression (Bakhoum and Cantley, 2018; Bakhoum et al., 2018). Given the high incidence of MN observed in cleavage-stage embryos from most mammals, it seems likely that cGAS-STING would serve as a surveillance mechanism for DNA release in this context as well, but this is still speculative (Figure 2B). Additional research suggested that the cGAS-STING pathway may also play a role in autophagy, senescence, and apoptosis (Yang et al., 2017; Yang et al., 2019; Zierhut et al., 2019), processes that also frequently occur during preimplantation development.

### DNA is eliminated from the embryo by multiple mechanisms at the blastocyst stage

Despite the presence of MN, CF, and/or aneuploidy, cleavage-stage embryos may continue to divide and still successfully reach the blastocyst stage. This suggests that embryonic chromosomal instability can be overcome, but the frequency and underlying mechanism(s) of such events are unknown. Our group demonstrated that chromosome-containing CFs which persisted throughout preimplantation embryogenesis are often expelled to the perivitelline space prior to blastocyst formation (Daughtry et al., 2019) (Figure 2D). In addition, we also observed that non-dividing blastomeres from the early cleavage-stage may be excluded to the blastocoel cavity during the morula-to-blastocyst transition. Following CNV analysis, we determined that these excluded blastomeres were highly chaotic, with multiple chromosome losses and gains, and immunostaining revealed abnormal nuclear morphology with extensive DNA damage (Daughtry et al., 2019). A previous report showed that E-cadherin expression is absent or altered in the excluded blastomeres of human embryos with no or abnormal compaction, suggesting a disruption in cell-cell adhesion (Alikani, 2005). Another study with reconstituted mouse embryos containing a mixture of control and hyperploid blastomeres demonstrated that the latter exhibited slower cell cycle progression and frequent DNA fragmentation beginning at the 16-cell stage (Pauerova et al., 2020). However, we observed selection against blastomeres as early as the 2- to 8-cell stage when embryos are most susceptible to chromosome mis-segregation events, which indicates that these previously observed changes were the consequence rather than the cause of aneuploidy. Nevertheless, the extruded fragments and blastomeres would presumably be left behind upon embryo hatching from the zona pellucida and prevented from participating in further development.

A recent study also showed that blastocyst expansion mechanically constrains the TE layer, causing nuclear budding and the shedding of DNA into the cytoplasm, and that this was exacerbated by biopsying 5–10 TE cells for preimplantation genetic testing of aneuploidy (Domingo-Muelas et al., 2023) (Figure 2D). Thus, similar to fragment removal (Halvaei et al., 2016), TE biopsy may provide no benefit especially if the biopsied cells are not representative of the embryo overall (Gleicher et al., 2017; Wu et al., 2021; Ren et al., 2022), or actually harmful for development by causing further DNA loss. Whether preferential allocation of aneuploid cells to the TE layer makes these cells more susceptible to DNA elimination even in the absence of TE biopsy remains to be determined, but preliminary evidence in human blastocysts suggests that there is no difference in the frequency of aneuploidy in relation to cell lineage by scDNA-seq (Chavli et al., 2024). However, the study did observe that complex chromosomal abnormalities are more commonly observed in TE cells than the inner cell mass (ICM). For this work, the TE and ICM were separated by biopsy prior to disaggregation into single cells, which can result in cross contamination between cell types due to cytoplasmic strings that connect the ICM to TE cells (Ebner et al., 2020; Joo et al., 2023). Thus, additional studies are needed to confirm each lineage by isolating both DNA and RNA from the same individual cell for scDNA-seq and gene expression (Macaulay et al., 2015; Macaulay et al., 2016), respectively.

## Conclusion and future directions

CIN and aneuploidy are remarkably common in preimplantation embryos, but difficult to accurately detect due to differences in the mechanism(s) of meiotic versus mitotic errors, extent of chromosomal mosaicism, and the response to chromosome mis-segregation through MN formation and the elimination of DNA by one of the above-described processes. Further work is needed to connect the intracellular dynamics of chromosome loss with measurable morphological events such as mitotic timing, MN formation, CF incidence, and blastomere asymmetry to noninvasively assess embryo potential. Ultimately, the goal will be to identify possible therapeutic targets to prevent or alleviate aneuploidy altogether, which may be difficult to accomplish if CIN is inherent to natural conception and aneuploidy occurs at a similar frequency *in vitro* and *in vivo*.
